# Supplementary material for: Deletion of wfs1 Impairs Oligodendrocyte Precursor Cells Dorsal Distribution and Myelination Through the wfs1-hmgcs1 Axis in Zebrafish
Source: Biology (Basel). 2026 Aug 16;15(16):1402. doi: 10.3390/biology15161402 (PMC13510151; doi:10.3390/biology15161402)
Supplement: Supplementary file 1 [file biology-15-01402-s001.zip › File S1-Transcriptome sequencing data and differentially expressed genes (DEGs) in wfs1-- zebrafish at 5 dpf/1.GO/wfs1KOvsWT/ALL/wfs1KOvsWT.all.GOcc_DAG.pdf]

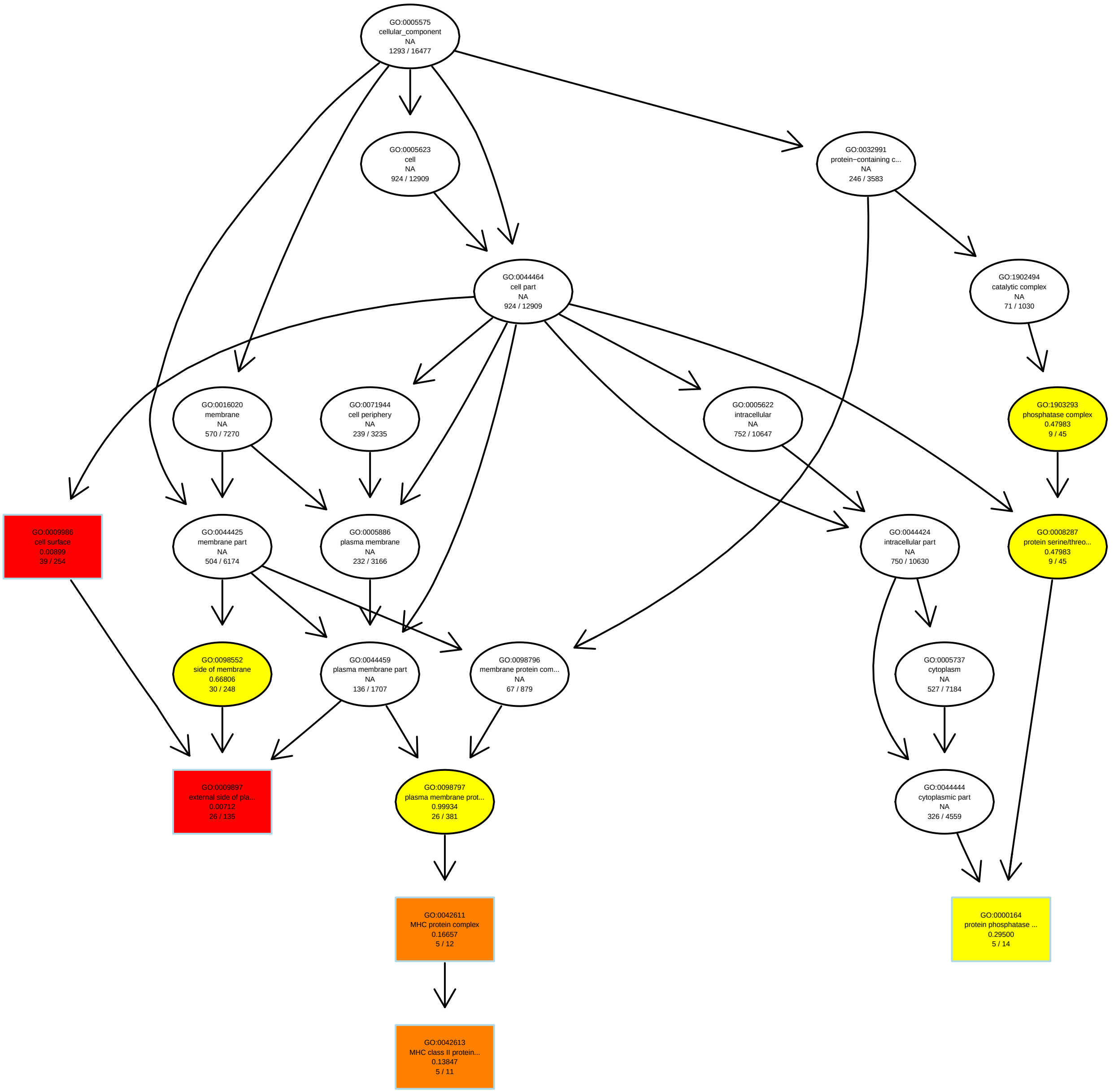
GO:0005575
cellular_component
NA
1293 / 16477
GO:0005623
cell
NA
924 / 12909
GO:0032991
protein−containing c...
NA
246 / 3583
GO:0044464
cell part
NA
924 / 12909
GO:1902494
catalytic complex
NA
71 / 1030
GO:0016020
membrane
NA
570 / 7270
GO:0071944
cell periphery
NA
239 / 3235
GO:0005622
intracellular
NA
752 / 10647
GO:1903293
phosphatase complex
0.47983
9 / 45
GO:0009986
cell surface
0.00899
39 / 254
GO:0044425
membrane part
NA
504 / 6174
GO:0005886
plasma membrane
NA
232 / 3166
GO:0044424
intracellular part
NA
750 / 10630
GO:0008287
protein serine/threo...
0.47983
9 / 45
GO:0098552
side of membrane
0.66806
30 / 248
GO:0044459
plasma membrane part
NA
136 / 1707
GO:0098796
membrane protein com...
NA
67 / 879
GO:0005737
cytoplasm
NA
527 / 7184
GO:0009897
external side of pla...
0.00712
26 / 135
GO:0098797
plasma membrane prot...
0.99934
26 / 381
GO:0044444
cytoplasmic part
NA
326 / 4559
GO:0042611
MHC protein complex
0.16657
5 / 12
GO:0000164
protein phosphatase ...
0.29500
5 / 14
GO:0042613
MHC class II protein...
0.13847
5 / 11
